# The complete chloroplast genome sequence *Holmskioldia sanguinea* retz., an ornamental plant of Lamiaceae

**DOI:** 10.1080/23802359.2020.1717392

**Published:** 2020-01-27

**Authors:** Yoonkyung Lee, Sangtae Kim

**Affiliations:** Department of Biology, Sungshin Women’s University, Seoul, Korea

**Keywords:** *Holmskioldia sanguinea*, chloroplast genome, genome skimming, ornamental plant, Scutellarioideae

## Abstract

*Holmskioldia sanguinea* Retz. (Lamiaceae) is a widely cultivated ornamental plant. As a first report in the genus, we present the complete chloroplast genome sequence of *H. sanguinea* using genome skimming of paired-end NGS data. The total genome size measures 153,273 bp in length containing four subregions: 84,693 bp of large single-copy region, 17,330 bp small single-copy region, and a pair of inverted repeat regions, measuring 25,625 bp. The genome contains 115 genes including 80 protein-coding genes, 4 rRNAs, and 31 tRNAs. Phylogenetic analysis showed that *H. sanguinea* is a sister to *Scutellaria* in the subfamily Scutellarioideae of Lamiaceae.

*Holmskioldia* Retz. is a monotypic genus and includes *H. sanguinea* Retz., which is distributed in southern Asia, Mauritius, Indonesia, and the West Indies (Atkins 1996). It is a scandent shrub with long brown-scarlet inflorescences. It has been unclear if this taxon is a member of Verbenaceae or Lamiaceae. However, recent molecular phylogenetic studies along with morphological studies suggest that *Holmskioldia* belong to Lamiaceae (Atkins 1996; Ryding 1995; Wagstaff and Olmstead 1997, 1998; Ryding 2007; Li et al. 2016), and is a sister to *Scutellaria* L. in the subfamily Scutellarioideae of Lamiaceae (Zhao et al. 2017; Safikhani et al. 2018).

We purchased *H. sanguinea* from the nursery and cultivated in the Sungshin University (N37°37′55.32″, E127°01′35.85″). A branch was used to prepare a voucher specimen (deposited in the herbarium of the Sungshin University; *Y. Lee 2019-001*, SWU). Total genomic DNA was extracted from fresh leaves using the GeneAll Plant SV Mini Kit (GeneAll Biotechnology Co. Ltd, Seoul, Korea) following the manufacturer’s protocol. The whole-genome sequencing was conducted with paired-end reads (100 bp in each length) using the BGISEQ-500 sequencer (BGI, Shenzhen, China).

A total of 34,772,388 reads (3.5 Gbp) were produced. To obtain the chloroplast (cp) genome sequence, we mapped each paired-end read against a cp genome from *Scutellaria insignis* Nakai (GenBank accession: NC_028533), a previously reported sister to *Holmskioldia*, using Geneious (v9.0.5; Kearse et al. 2012) with the ‘medium-low sensitivity option’. Subsequently, the quality of consensus sequences and their mapping condition were examined visually. Six specific primer pairs in the regions of *petG*–*psaJ*, *psbB*, *rpl23*–*ycf2*, *rpoA*, *rrn16*, and *rrn16*–*trnA*-UGG were designed (sequences not shown) for filling gaps. The PCR and Sanger sequencing were conducted with the condition from Song et al. (2019). The cp genome was annotated using GeSeq (Tillich et al. 2017). The annotated genome was compared with a cp genome of *S. insignis* in the alignment generated by MAFFT (v7.308; Katoh and Standley 2013) module in the Geneious (v9.0.5; Kearse et al. 2012).

The complete cp genome of *H. sanguinea* is 153,273 bp in length (GenBank accession: MN227130), containing a large single-copy (LSC) of 84,693bp, a small single-copy (SSC) of 17,330 bp, and a pair of inverted repeat (IR) regions of 25,625 bp. The genome includes 115 genes comprising 80 protein-coding genes, 4 rRNA genes, and 31 tRNA genes.

For the phylogenetic analysis, 11 representative cp genomes were selected from each subfamilies of Lamiaceae based on the phylogenetic information obtained from Li et al. (2016) (Figure 1). The phylogenetic tree (Figure 1) showed that *H. sanguinea* belongs to a clade of subfamily Scutellarioideae and is a sister to *Scutellaria*.

**Figure 1.**
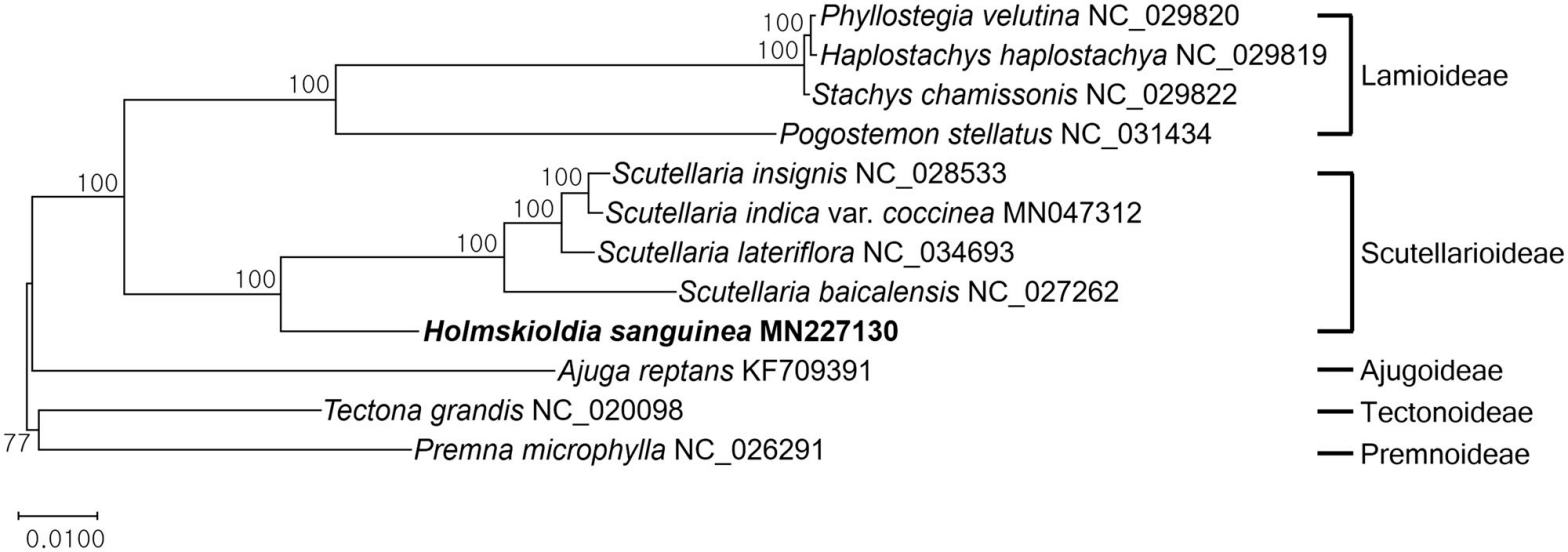
A maximum-likelihood tree based on a cp genome of *H. sanguinea* and 11 subfamilial cp genomes in Lamiaceae using raxmlGUI (v.1.5; Silvestro and Michalak 2012) with 1,000 bootstrap replications. The GTR + Gamma + I model was selected as the best model using a module of the model test in MEGA7 (Kumar et al. 2016). Numbers above the node indicate bootstrap values.

In this study, we report the complete cp genome sequence from *H. sanguinea*, a widely cultivated garden plant. This study will provide basic information elucidating the phylogeny and evolution of taxa in the subfamily Scutellarioideae. It also provides outgroup information for cp genome studies of *Scutellaria*, which is one of the largest genera in the Lamiaceae.
